# Design and implementation of a Wireless Power Transfer with double-sided LCC compensation for a farming robot

**DOI:** 10.1371/journal.pone.0359107

**Published:** 2026-09-28

**Authors:** Aryadharma Sudhartio, Marojahan Tampubolon, Gervasius Geovan, M. B. Nugraha, Ahmad Syahril Muharom

**Affiliations:** 1 Electrical Engineering Study Program, Universitas Multimedia Nusantara, Kabupaten Tangerang, Banten, Indonesia; 2 Department of Electrical Engineering, National Central University, Taoyuan, Taiwan; 3 Department of Electrical and Electronic Engineering, Mie University, Tsu, Japan; University of Cagliari, ITALY

## Abstract

Farming robots are used nowadays to increase farming efficiency and production. In this paper, the Wireless Power Transfer (WPT) is proposed to be used as the enabler technology to support the autonomous characteristics of the robot. A low input voltage supplied from a solar panel is assumed as the source for the WPT system in this work. The mathematical modeling, design, and hardware implementation are discussed in this paper. The experimental results of WPT with double-sided LCC compensation maintained its efficiency to a certain distance with output power 5–76 W under the variation of coupling coefficient from 0.18 to 0.05. The peak efficiency obtained from the experiment was 69.19%. In addition, the study also showed that the output voltage of WPT can be regulated at 24V by changing the switching frequency of the inverter.

## Introduction

Agriculture has a significant role in a country’s growth [1] due to the increasing needs of the crops to feed the population over time. This demand pushes the agriculture technology for improvement, especially the farming sector [2,3]. Farming industries have developed along with the Industrial Revolution since the 17th century. In Industrial Revolution 4.0, the farming sector has reached more advanced tools because of the digital technology integration [4]. One of the technology developments in farming is smart farming.

One part of smart farming is using an autonomous robot, or an agriculture robot, to operate in hard-to-access areas [1,5]. The robot used in farming sector have a considerable risk to be contaminated with dust, dirt, mud, or another filth, resulting in disturbance of power charging [6], to optimize charging in farming robot, a large capacity battery can be used, however this method can increase the robot size, weight, and affect robot performance [7], so another solution are needed for a farming robot charging system.

A practical, autonomous, and efficient charging system for a farming robot can be achieved using Wireless Power Transfer (WPT) [8,9]. With WPT, the charging process can work without problem during rainy days [10], even when the farming robots are contaminated by dirt [11–13], and does not interfere with the work process. The resonant inductive coupling method is used for the WPT, using coils on the transmitter and receiver sides with a compensation circuit to achieve resonance frequency and higher efficiency [14,15].

Wireless transferred energy using mutual inductance between the transmitter and receiver coils. The leakage inductance is significant due to the coil gap, misalignment, and tilt. Use of a compensation circuit to compensate for the leakage inductance is a must to make power transfer more efficient [16,17]. Leakage inductance compensation is done by adding a capacitor. Basic types of compensation circuits are using a capacitor connected in series (SS), parallel (PP), series-parallel (SP), or parallel-series (PS) [18–20]. An SS compensation circuit is simple and low-cost, but its performance is highly dependent on the mutual inductance and load dynamics, which makes it require a more complex control system [21–23]. The SP-type compensation is more suitable for a current-source application, resulting in a better efficiency for an asymmetrical coil case in which the transmitter side is bigger [24]. A more advanced compensation circuit is achieved by adding an inductor or an additional capacitor to the basic compensation to achieve different system characteristics. One of the advanced compensation circuits is a double-sided LCC [25,26]. With double-sided LCC, the system can achieve zero-voltage switching (ZVS) by adjusting the compensation component parameter [27,28]. Another advantage of double-sided LCC compensation is its robustness against misalignment, slope, and coil gap, resulting in more stable efficiency. Considering the environment for farming robots that are subject to the presence of dirt, gravel, and uneven surfaces, the double-sided LCC compensation is selected [29].

## Working principle and system modeling

The design of WPT utilized here is based on the inductive coupling model, which means the power will be transferred using the changing magnetic field in the transmitter coil that induces a voltage in the receiver coil. The WPT system can be illustrated by a circuit diagram as shown in Fig 1. The input voltage of the system is a low DC voltage (24 V), which is then converted into an AC voltage using a full bridge inverter by Q1-Q4 MOSFETs. After the inverter circuit passes, then U1 has become a square wave AC voltage. The compensation method to cancel out leakage induction in the designed system coil uses double-sided LCC compensation, consisting of 1 inductor and two capacitors connected in series and parallel, both on the transmitter and receiver sides. After the power is successfully transferred, the voltage is rectified to DC using a full bridge rectifier by D1-D4 before being used by the load (RL).

**Fig 1:**
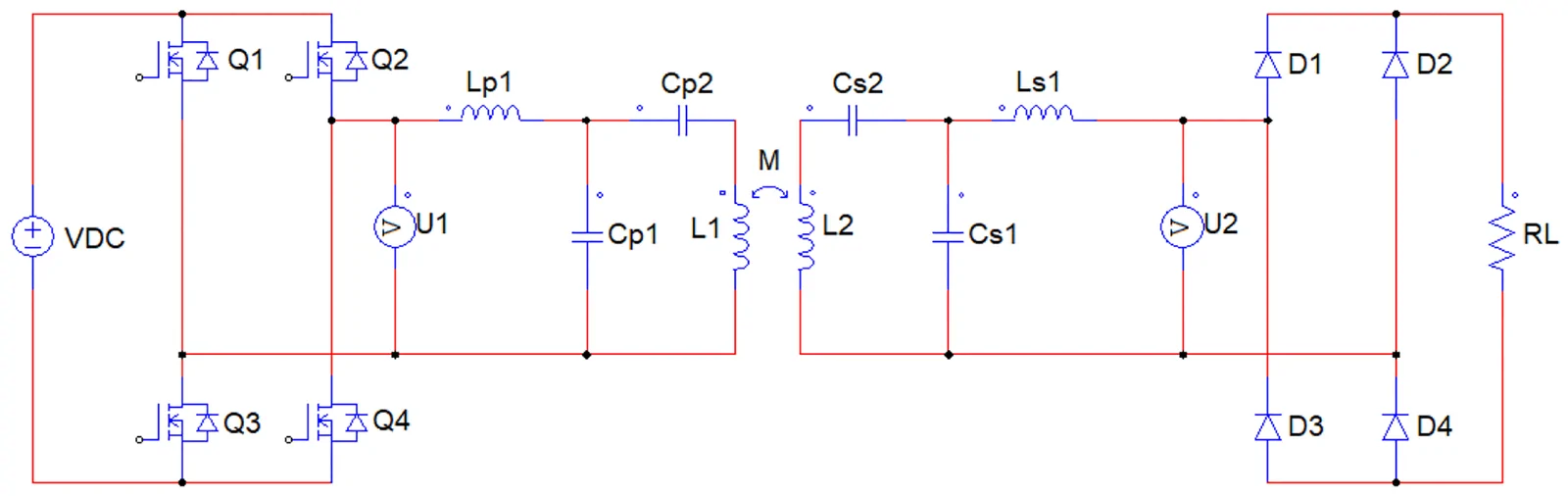
Double-sided LCC wireless power transfer circuit diagram.

MOSFETs in full-bridge inverters operate with a gate signal given by an STM32 microcontroller with a 50% duty cycle. Dead time is used in the gate signal to ensure there is no short circuit between the transitions of each MOSFET pair. A bootstrap circuit and gate resistor are used to generate a good gate signal to turn the MOSFETs on and off effectively.

First harmonic approximation is used to model the system to assume the inverter voltage is a pure sine wave AC signal expressed by:


$$\begin{array}{c} \begin{array}{c} V_{S(peak)}=\;\frac{\text{4}}{\pi }\times V_{DC} \end{array} \end{array}$$ (1)

The load and full bridge rectifier circuits are then simplified to an equivalent circuit with Eq. 2 to make system modelling easier. After the simplification, the WPT system circuit now only consists of resistors, inductors, and capacitors, as shown in Fig 2.

**Fig 2:**
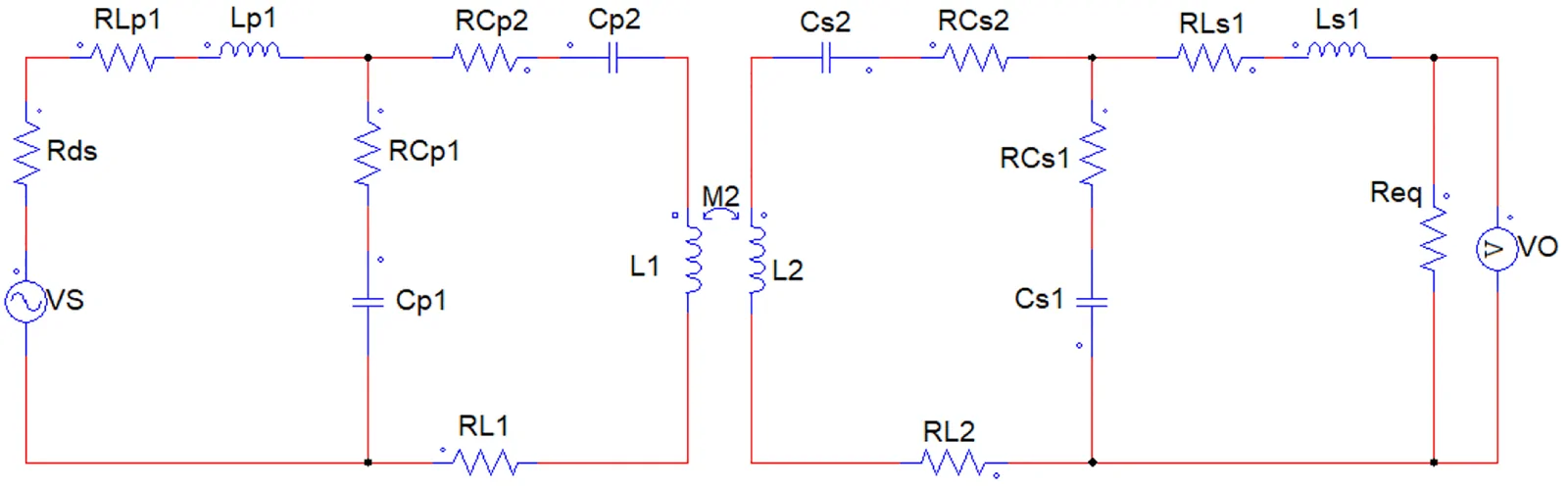
Simplified WPT system.


$$\begin{array}{c} \begin{array}{c} R_{eq}=\;\frac{V_{in(AC)rms}}{I_{Srms}}=\;\frac{\frac{\text{2}\sqrt{\text{2}}}{\pi }\times V_{Out}}{\frac{\pi }{\text{2}\sqrt{\text{2}}}\times I_{Out}}=\;\frac{\text{8}}{\pi^{\text{2}}}\times R_{L} \end{array} \end{array}$$ (2)

The WPT system can then be simplified further using equivalent impedance, including the parasitic resistance of the inductor, capacitor, and coil. The equivalent impedance of the system is expressed by Eqs 3–9, resulting in matrix Eq 10.


$$\begin{array}{c} \begin{array}{c} a=R_{DS}+\;sL_{p\text{1}}+R_{Lp\text{1}}+\frac{\text{1}}{sC_{p\text{1}}}+R_{cp\text{1}} \end{array} \end{array}$$ (3)

$$\begin{array}{c} \begin{array}{c} b=-\left(\frac{\text{1}}{sC_{p\text{1}}}+R_{cp\text{1}}\right) \end{array} \end{array}$$ (4)

$$\begin{array}{c} \begin{array}{c} c=sL_{\text{1}}+R_{L\text{1}}+\frac{\text{1}}{sC_{p\text{2}}}+R_{cs\text{2}}+\frac{\text{1}}{sC_{p\text{1}}}+R_{cs\text{1}} \end{array} \end{array}$$ (5)

$$\begin{array}{c} \begin{array}{c} d=-sM \end{array} \end{array}$$ (6)

$$\begin{array}{c} \begin{array}{c} e=sL_{\text{2}}+R_{L\text{2}}+\frac{\text{1}}{sC_{s\text{2}}}+R_{cs\text{2}}+\frac{\text{1}}{sC_{s\text{1}}}+R_{cs\text{1}} \end{array} \end{array}$$ (7)

$$\begin{array}{c} \begin{array}{c} f=-\left(\frac{\text{1}}{sC_{s\text{1}}}+R_{cs\text{1}}\right) \end{array} \end{array}$$ (8)

$$\begin{array}{c} \begin{array}{c} g=sL_{s\text{1}}+R_{Ls\text{1}}+\frac{\text{1}}{sC_{s\text{1}}}+R_{cs\text{1}}+R_{eq} \end{array} \end{array}$$ (9)

$$\begin{array}{c} \begin{array}{c} \left[\begin{array}{c} V_{S}\\ \text{0}\\ \text{0}\\ \text{0} \end{array}]=\;[\begin{array}{cccc} a & b & \text{0} & \text{0}\\ b & c & d & \text{0}\\ \text{0} & d & e & f\\ \text{0} & \text{0} & f & g \end{array}]\;[\begin{array}{c} I_{Lf\text{1}}\\ I_{\text{1}}\\ I_{\text{2}}\\ I_{Lf\text{2}} \end{array}\right] \end{array} \end{array}$$ (10)

Obtained matrix of system impedance can be derived to obtain the system transfer function, resulting in Eq 11.


$$\begin{array}{c} \begin{array}{c} \frac{V_{O}}{V_{S}}=\;\frac{b\times d\times f}{-b^{\text{2}}\times f^{\text{2}}+e\times g\times b^{\text{2}}+a\times g\times d^{\text{2}}+a\times c\times f^{\text{2}}-a\times c\times e\times g}\times R_{eq} \end{array} \end{array}$$ (11)

The resulting system transfer function can be plotted in MATLAB to obtain the Bode plot, as shown in Figs 3 and 4, to observe changes in the system response with switching frequency and the effects of load and coupling coefficient variation on the output voltage gain. Generated Bode plots show us that the system can achieve the desired output voltage by changing the switching frequency. This information means that the system can be controlled by frequency modulation. Plot by varying output load and coupling coefficient indicates that output voltage gain is reduced inversely proportional to coupling coefficient and output load.

**Fig 3:**
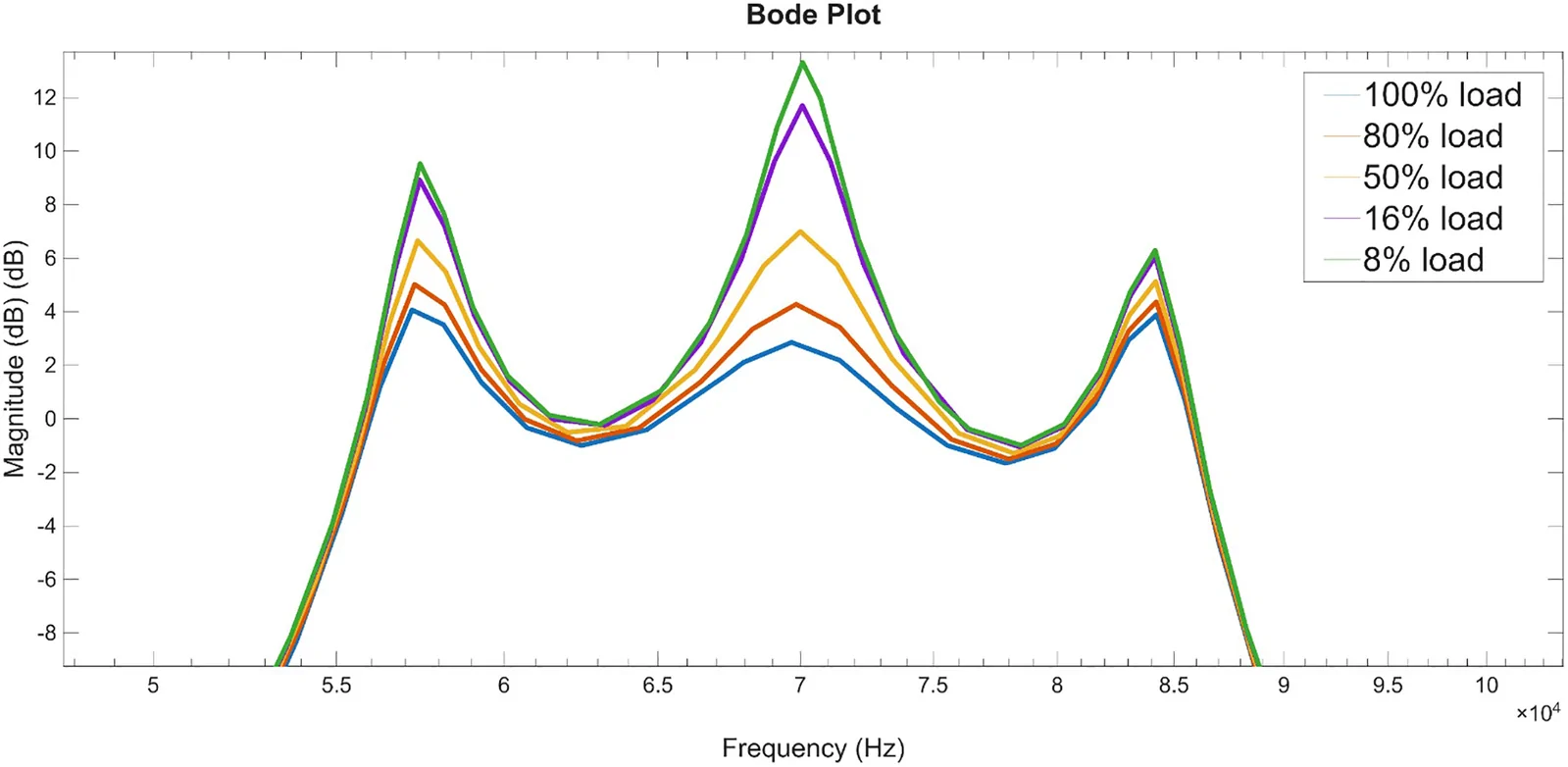
Bode plot with load variations at coupling coefficient = 0.2.

**Fig 4:**
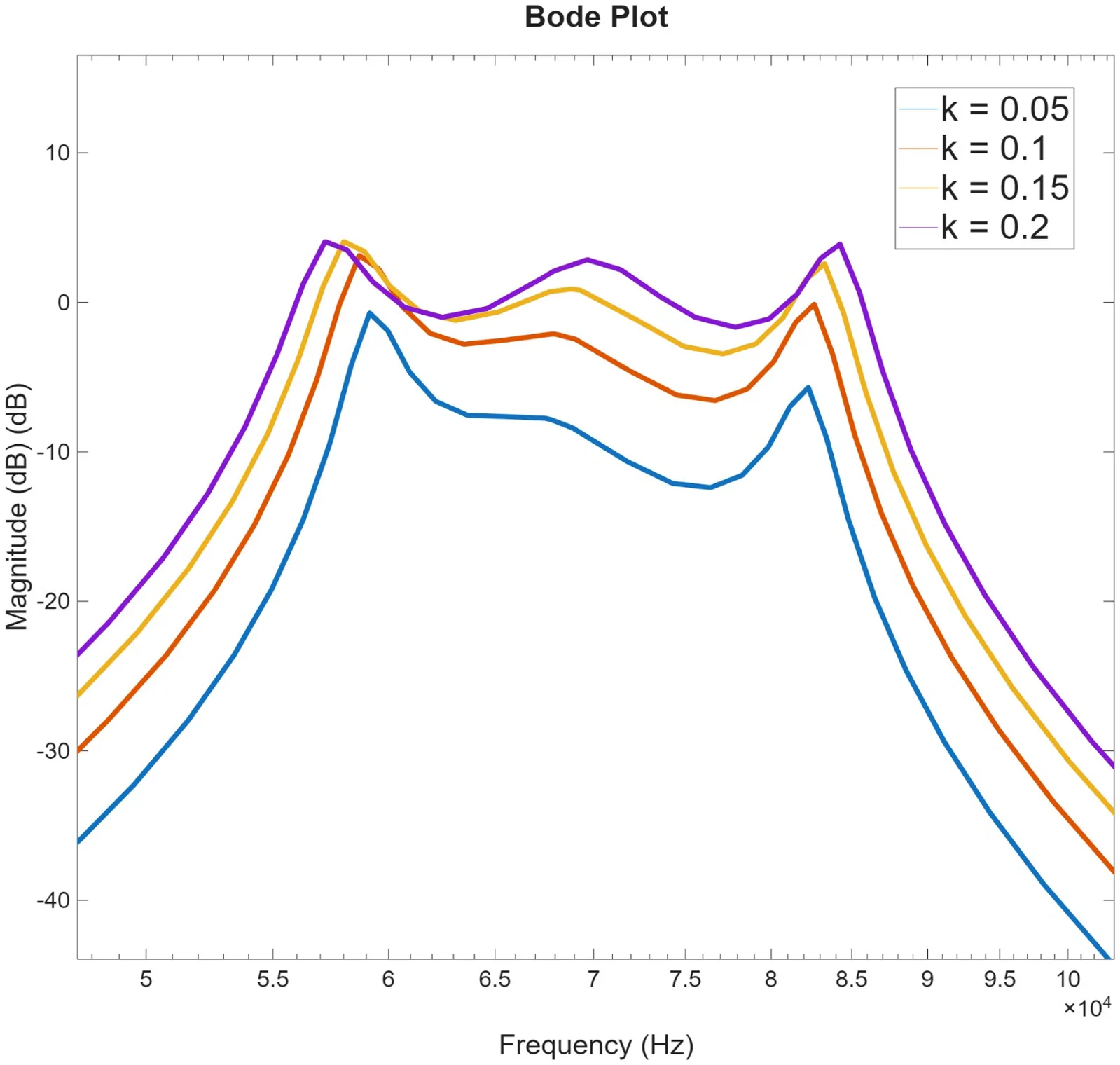
Bode plot with coupling coefficient variation at full load.

## Key components consideration

In order to design a WPT system that operates more efficiently, the system must operate at the resonance frequency where capacitive and inductive reactance cancel each other, resulting in a near unity power factor. Resonance frequencies are determined by a coupled inductor in a coil, an inductor, a capacitor, and a compensation circuit. First, coil and inductor are defined, and the capacitor capacitance value can be calculated by Eqs. 12 and 13.


$$\begin{array}{c} \begin{array}{c} C_{p\text{1}}=C_{s\text{1}}=\;\frac{\text{1}}{\omega^{\text{2}}\;\times \;L_{p\text{1}}} \end{array} \end{array}$$ (12)

$$\begin{array}{c} \begin{array}{c} C_{p\text{2}}=C_{s\text{2}}=\;\frac{\text{1}}{\omega^{\text{2}}\;\times \;\left(L_{\text{1}}-\;L_{p\text{1}}\right)} \end{array} \end{array}$$ (13)

Table 1 lists the parameter values used in the components of the designed WPT system.

**Table 1:** Designed system parameters value.

Parameters	Value
V_in_ (V)	24
Switching frequency (kHz)	60-80
Lp_1_ (µH)	15
Cp_1_ (µF)	0.3
Cp_2_ (µF)	0.03
L_1_ (µH)	200
L_2_ (µH)	200
Cs_1_ (µF)	0.3
Cs_2_ (µF)	0.03
Ls_1_ (µH)	15
V_out_ (V)	24
I_out_ (A)	3

## Hardware implementation

The designed WPT system mainly consists of 3 hardware: transmitter PCB, Transmitter & Receiver coils, and receiver PCB. Subsystems such as the inverter and compensation are located on the transmitter PCB, while the full-bridge rectifier is on the receiver PCB; compensation and driver circuits are used in both. The coils used for power transfer consist of circularly wound litz wire with dimensions of 20x20 cm, and ferromagnetic cores are used to guide the magnetic field and increase the mutual inductance. The designed hardware used is shown in Fig 5. The power source for the real field application is solar panels, with a typical system that includes Maximum Power Point Tracking (MPPT) inserted between the solar panel and the Battery. However, for the laboratory prototype, a DC power supply was used to simulate the battery in the field application. System testing starts with subsystem evaluation, inverter output voltage, compensation performance, and coupling coefficient under various misalignment and coil gap conditions. After all subsystems have functioned properly, the system output is tested with variations of load, misalignment, coil gap, and slope.

**Fig 5:**
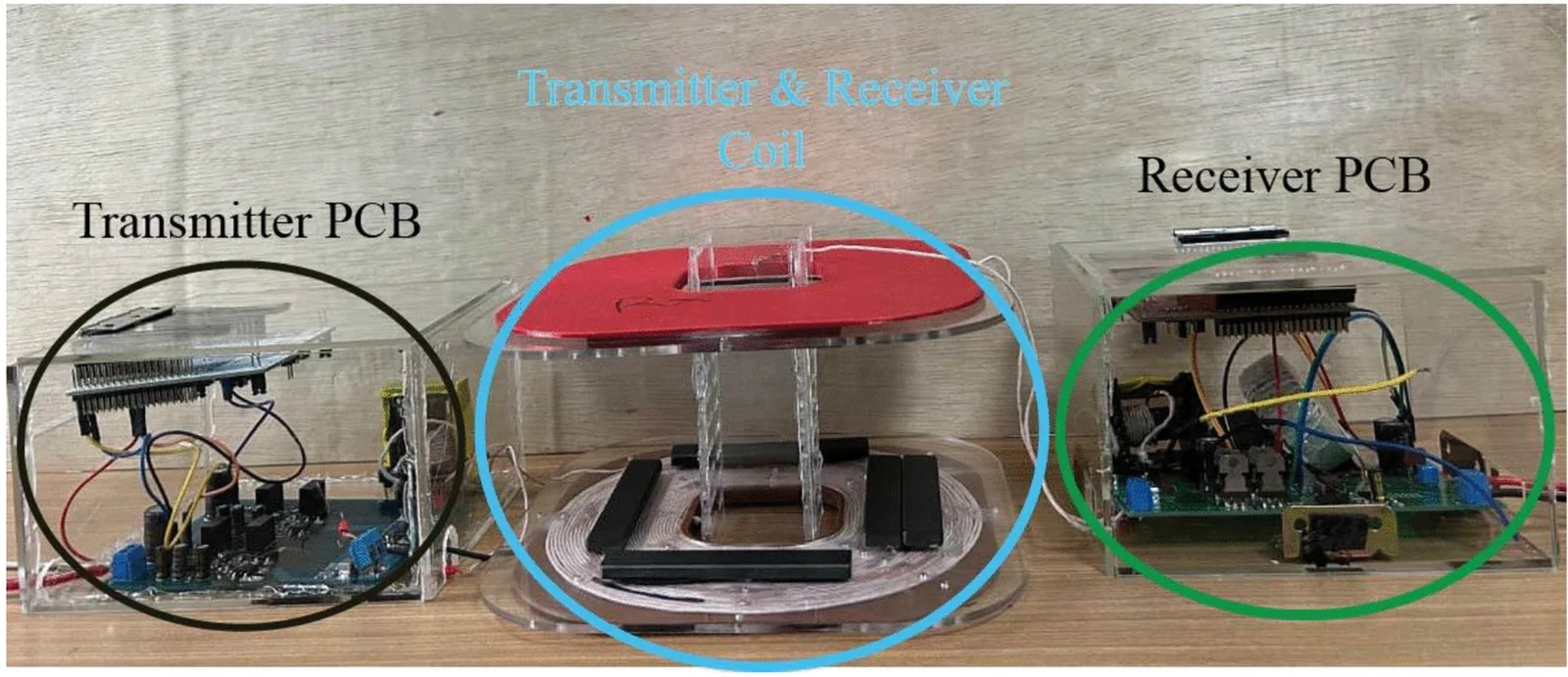
Developed hardware for the WPT system.

## Results and analysis

The inverter output voltage is shown in Fig 6, indicating the circuit has successfully converted a DC voltage source into a square wave AC voltage. The Gate (G) and the Drain (D) voltage waveforms are also observed to show the condition of switching transition, as shown in Fig 7. It shows that at the turn-on transition, the D-S voltage has reached zero voltage before the G-S voltage rises. In this case, the G-S voltage is the representation of the Drain-Source Current (I_DS_) since the current can flow only if the MOSFET is on. Therefore, at the transition zone, the D-S voltage multiplication to I_DS_ is close to zero. This phenomenon indicates that the inverter is operating in zero voltage switching (ZVS) mode, resulting in a reduction of switching loss of the MOSFET. A compensation circuit is used to cancel out the effect of leakage induction to make the load seem as fully resistive, with a near unity power factor of transferred energy, resulting in efficient power transfer. Voltage and current output of the compensation circuit in Fig 8 demonstrate that the designed double-sided LCC compensation can realize a near unity power factor on the receiver side.

**Fig 6:**
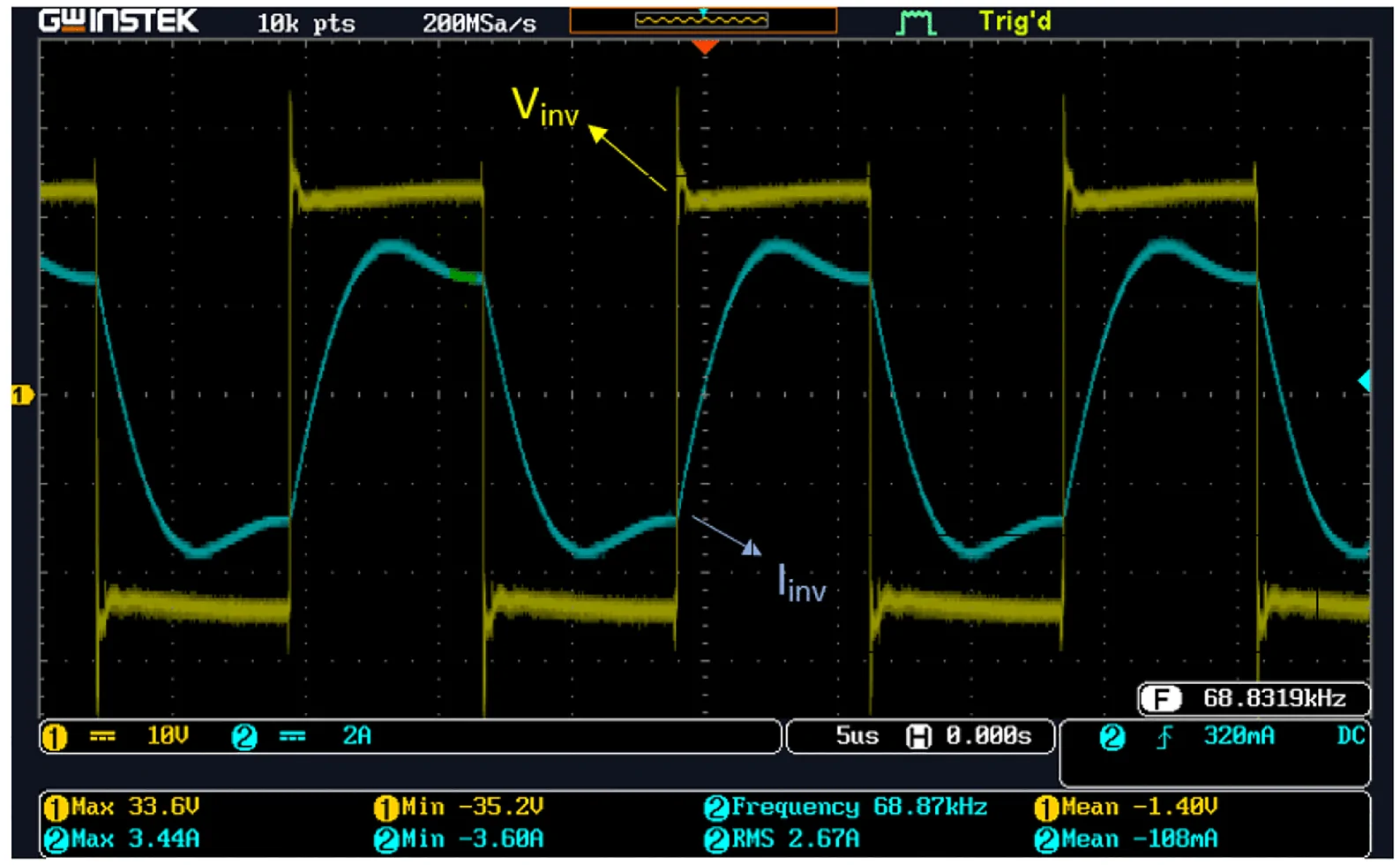
Inverter output voltage.

**Fig 7:**
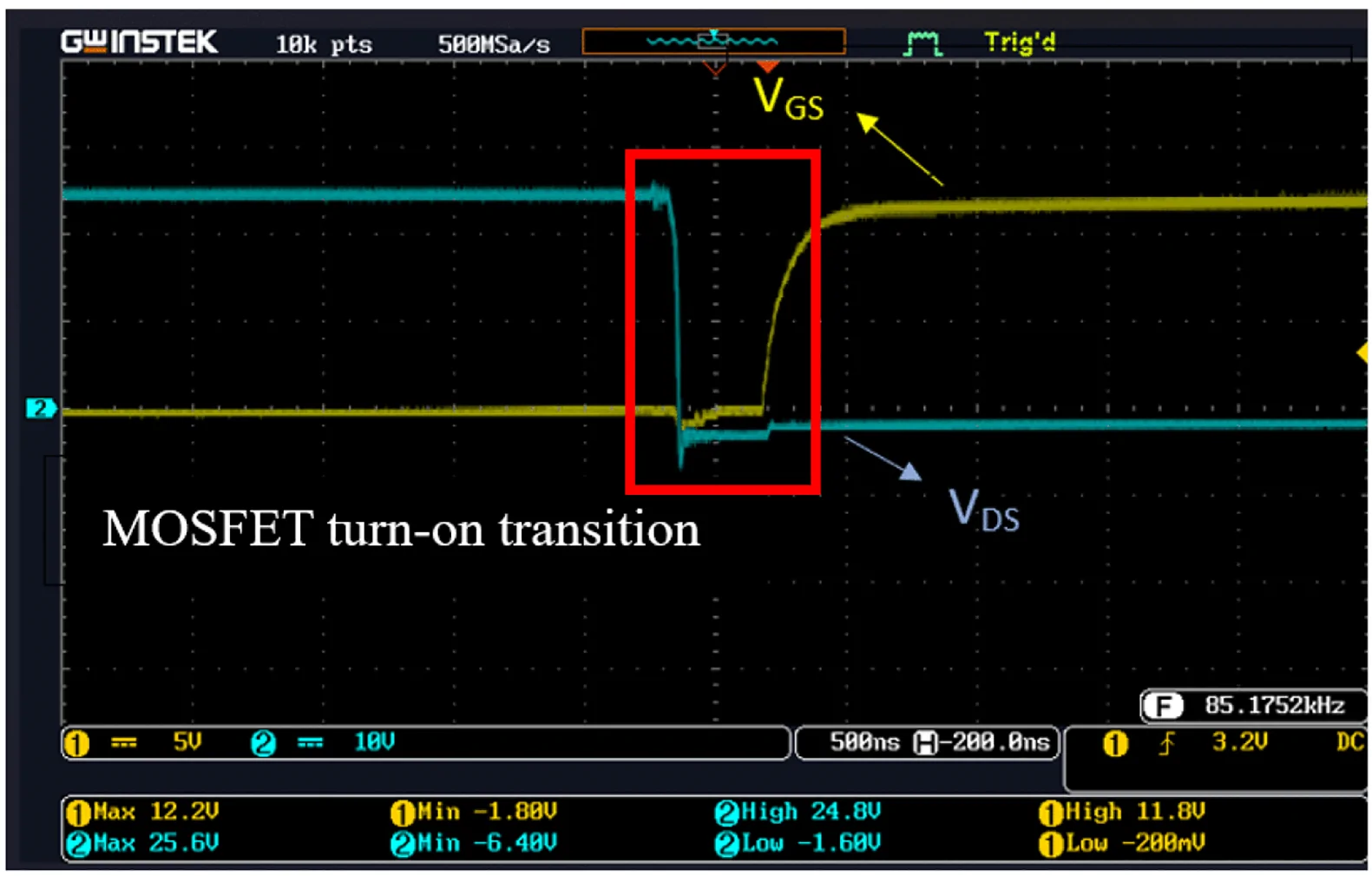
Turn-on transition of the inverter MOSFET.

**Fig 8:**
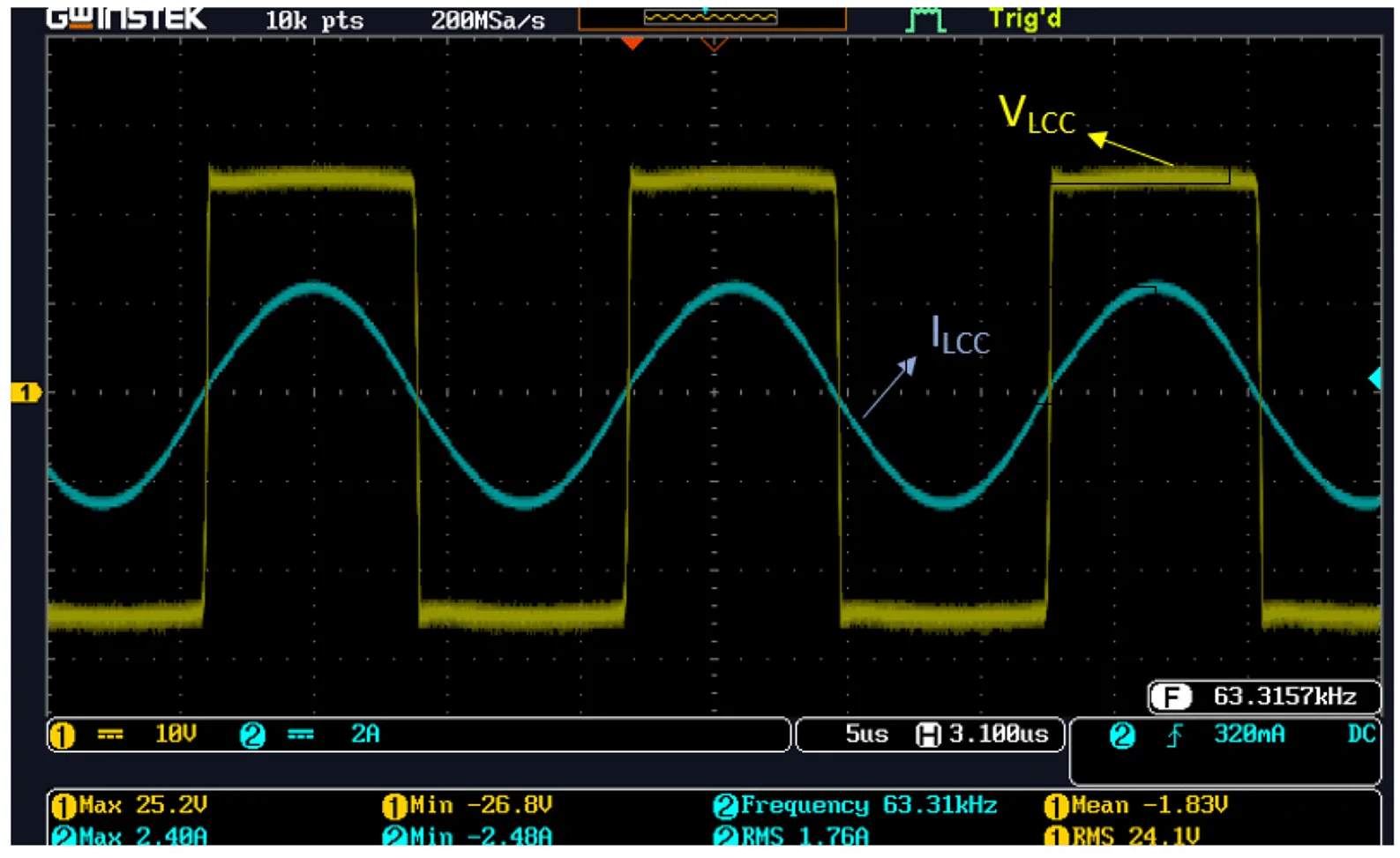
Double-sided LCC compensation output voltage and current.

The transmitter and receiver coils in the WPT system can be shifted, resulting in the coupling coefficient reduction. The coupling coefficient is a constant that indicates how much mutual inductance can be used. Table 2 and Fig 9 show the coupling coefficient value under the effect of lateral misalignment and vertical gap between transmitter and receiver coil.

**Table 2:** Coupling coefficient with varying misalignment and coil gap.

Gap (cm)	Coupling Coefficient	Misalignment (cm)
4	0,181	0
4	0.174	2
4	0.147	4
4	0.114	6
5	0.143	0
5	0.131	2
5	0.110	4
5	0.085	6
6	0.120	0
6	0,113	2
6	0.097	4
6	0.079	6
7	0.098	0
7	0.093	2
7	0.081	4
7	0.066	6
8	0.083	0
8	0.079	2
8	0.069	4
8	0.057	6

**Fig 9:**
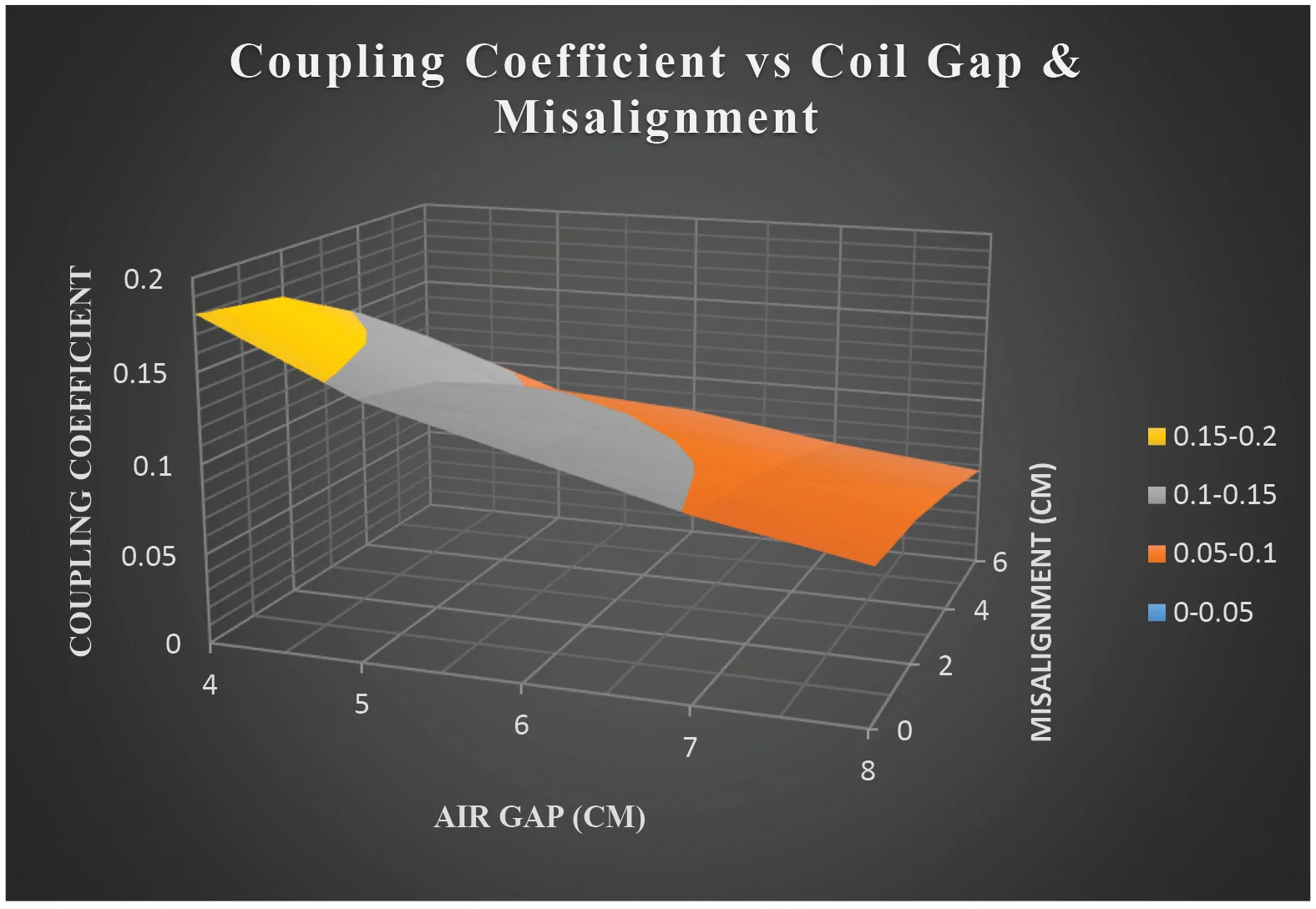
Coupling coefficient with varying misalignment and coil gap.

The system’s outputs are tested under conditions of varying loads, misalignment, gap, and coil slope with respect to each other. Tables 3 and 4 show the testing data at 16Ω resistor (50% load) and 8Ω resistor (100% load), respectively. The vertical gap variations are from 4 to 8 cm and lateral misalignment variation up to 6 cm. It should be noted that the data presented for each condition in this table is the average value for five repetitive data collections of the same condition. The presented data show a variation of switching frequencies, which are manually tuned to provide the output voltage close to 24V as the target at the respective condition. The output voltage of the system at this condition can be maintained around 24V with a range and variance of 1V and 0.063 for 16Ω load and 0.92V and 0.040 for 8Ω load. It shows that a regulated output voltage can be achieved with variable frequency control. This data also shows the impact of the vertical gap and lateral misalignment on the efficiency. The efficiency tends to decrease slightly when the vertical gap increases. However, the variation of lateral misalignment does not always result in a decrease in efficiency. The maximum efficiency obtained for 16Ω load is 69.19% at a 4 cm vertical gap, 2 cm lateral misalignment, and no angular misalignment. For 8Ω load, the maximum efficiency obtained was 64.6% at 6 cm vertical gap without lateral and angular misalignment.

**Table 3:** System output with 16Ω load and no angular misalignment.

Vertical Gap (cm)	Coupling Coef.	Lat. Misalignment (cm)	Freq. (kHz)	V_out_(V)	P_in_(W)	P_out_(W)	Eff (%)
4	0.18	0	69.5	24.3	56.6	38.5	68.0
4	0.17	2	69.3	23.4	51.4	35.5	69.2
4	0.15	4	67.8	23.8	54.7	36.2	66.2
4	0.11	6	65.9	24.0	59.0	36.4	61.7
5	0.14	0	68.0	23.9	56.2	36.3	64.7
5	0.13	2	67.5	24.3	56.6	38.4	67.8
5	0.11	4	66.6	23.9	58.1	36.4	62.7
5	0.09	6	65.1	24.4	65.8	38.8	58.9
6	0.12	0	66.6	24.2	59.5	37.3	62.7
6	0.11	2	66.1	24.0	60.5	36.5	60.4
6	0.10	4	65.2	24.1	65.8	37.1	56.5
6	0.08	6	64.1	23.6	75.4	35.9	47.7
7	0.10	0	65.5	23.8	62.9	36.2	57.6
7	0.09	2	65.2	24.2	65.3	37.7	57.7
7	0.08	4	64.6	23.9	70.1	36.6	52.3
7	0.07	6	63.7	23.9	85.4	36.6	42.9
8	0.08	0	64.5	24.2	74.4	36.9	49.6
8	0.08	2	64.4	24.1	75.4	37.4	49.6
8	0.07	4	63.9	23.7	83.5	36.2	43.3
8	0.06	6	63.3	23.6	103.2	36.0	34.9

**Table 4:** System output with 8Ω load and no angular misalignment.

Vertical Gap (cm)	Coupling Coef.	Lat. Misalignment (cm)	Freq. (kHz)	V_out_(V)	P_in_(W)	P_out_(W)	Eff (%)
4	0.18	0	68.3	23.76	113.76	71.9	63.2
4	0.17	2	67.9	24.08	115.20	73.2	63.5
4	0.15	4	66.6	24.50	118.08	74.6	63.2
4	0.11	6	61.9	24.30	121.44	73.9	60.8
5	0.14	0	66.9	24.20	115.68	73.6	63.6
5	0.13	2	66.5	24.10	115.20	73.3	63.6
5	0.11	4	65.4	24.04	116.16	73.1	62.9
5	0.09	6	62.5	23.98	124.80	72.9	58.4
6	0.12	0	65.5	24.00	112.80	72.9	64.6
6	0.11	2	65.0	24.00	115.20	73.0	63.3
6	0.10	4	62.6	23.88	122.40	72.5	59.3
6	0.08	6	62.5	23.94	143.04	72.8	50.9
7	0.10	0	62.5	24.08	125.76	73.2	58.2
7	0.09	2	62.6	24.14	126.72	73.4	57.9
7	0.08	4	62.6	24.08	134.40	73.2	54.5
7	0.07	6	62.4	23.58	151.20	71.3	47.2
8	0.08	0	62.5	24.10	142.08	73.2	51.5
8	0.08	2	62.7	24.02	135.84	73.0	53.8
8	0.07	4	62.5	24.06	153.60	73.1	47.6
8	0.06	6	62.3	23.64	172.80	71.5	41.4

The other measurements were done for various angular misalignments, combined with the lateral misalignments at various loads and the fixed vertical gap of 8 cm. These combinations simulate the best and the worst-case scenarios in the practical application at an 8 cm vertical gap. The measurement results in Table 5 show that the smallest efficiency is 8.6%, and the maximum efficiency is 73.2%. As expected, the output power decreases at the maximum lateral misalignment. However, for the angular misalignment, the output power has no clear trend, but tends to increase when the angular misalignment increases. The possible cause of this phenomenon is the closer proximity of one side of the receiver coil to the transmitter coil as the transmitter tilts.

**Table 5:** System output at 8 cm vertical gap, various angular misalignments.

Angular misalignment (deg)	Output Power (W)				Efficiency (%)			
	R = 100Ω	R = 50Ω	R = 16Ω	R = 8Ω	R = 100Ω	R = 50Ω	R = 16Ω	R = 8Ω
**8 cm vertical gap, no lateral misalignment**								
0	6.1	11.9	36.9	73.2	19.9	29.6	49.6	51.5
15	5.6	11.4	36.6	72.7	24.2	37.6	54.9	59.2
30	6.2	11.5	36.5	72.7	28.7	38.5	57.6	62.3
**8 cm vertical gap, 6 cm lateral misalignment**								
0	6.9	12.4	36.0	71.5	12.0	19.6	34.9	41.4
15	5.4	11.5	36.0	66.8	10.94	22.8	42.6	44.9
30	6.1	11.3	36.5	52.2	8.6	14.2	30.6	36.9

The efficiency curves for maximum and minimum coupling coefficient are shown in Figs 10 and 11. Maximum efficiency of the designed system is achieved at 50% load condition, reaching 69.13%, and the worst efficiency is under light load condition (8%) and minimum coupling coefficient.

**Fig 10:**
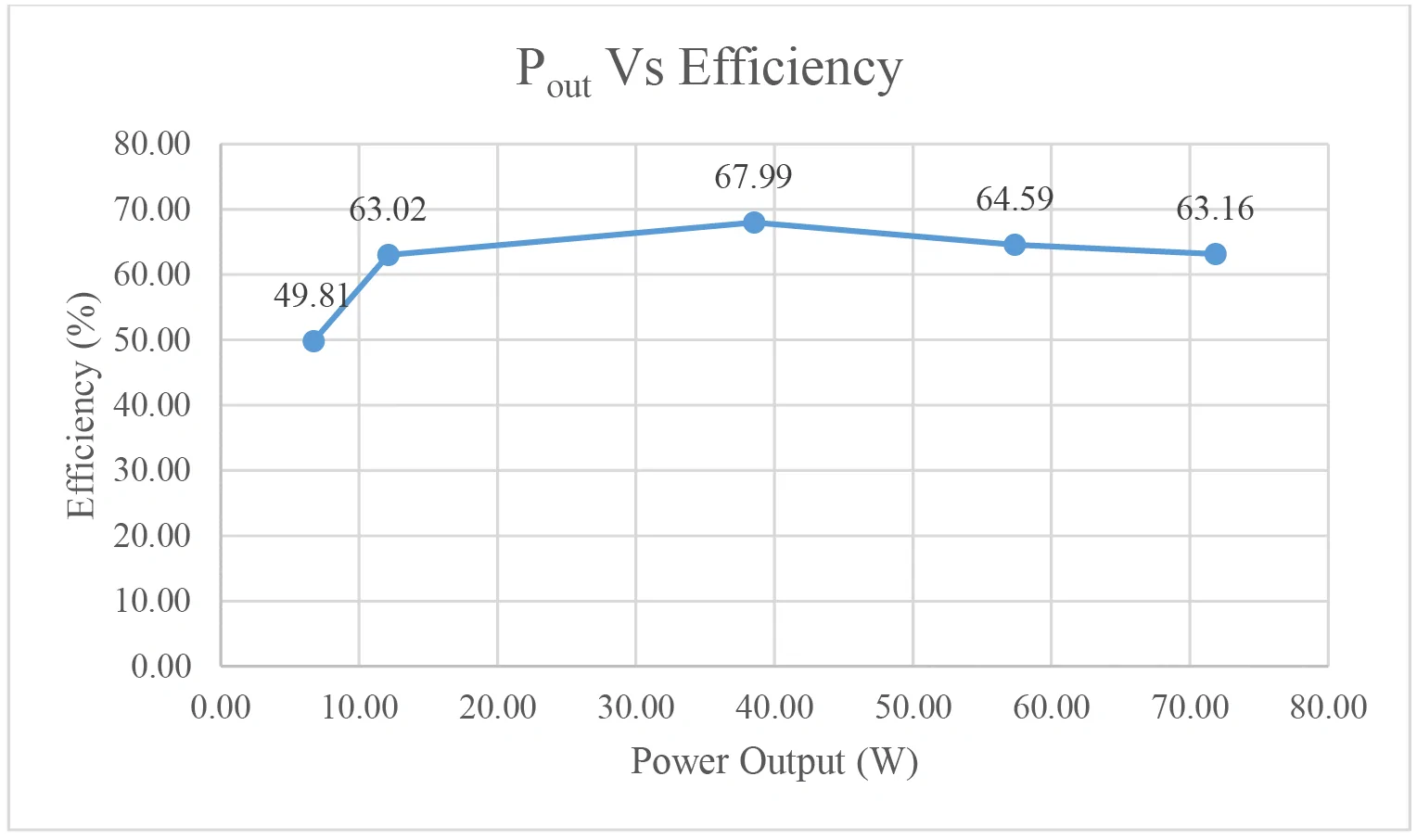
WPT System efficiency at maximum coupling coefficient.

**Fig 11:**
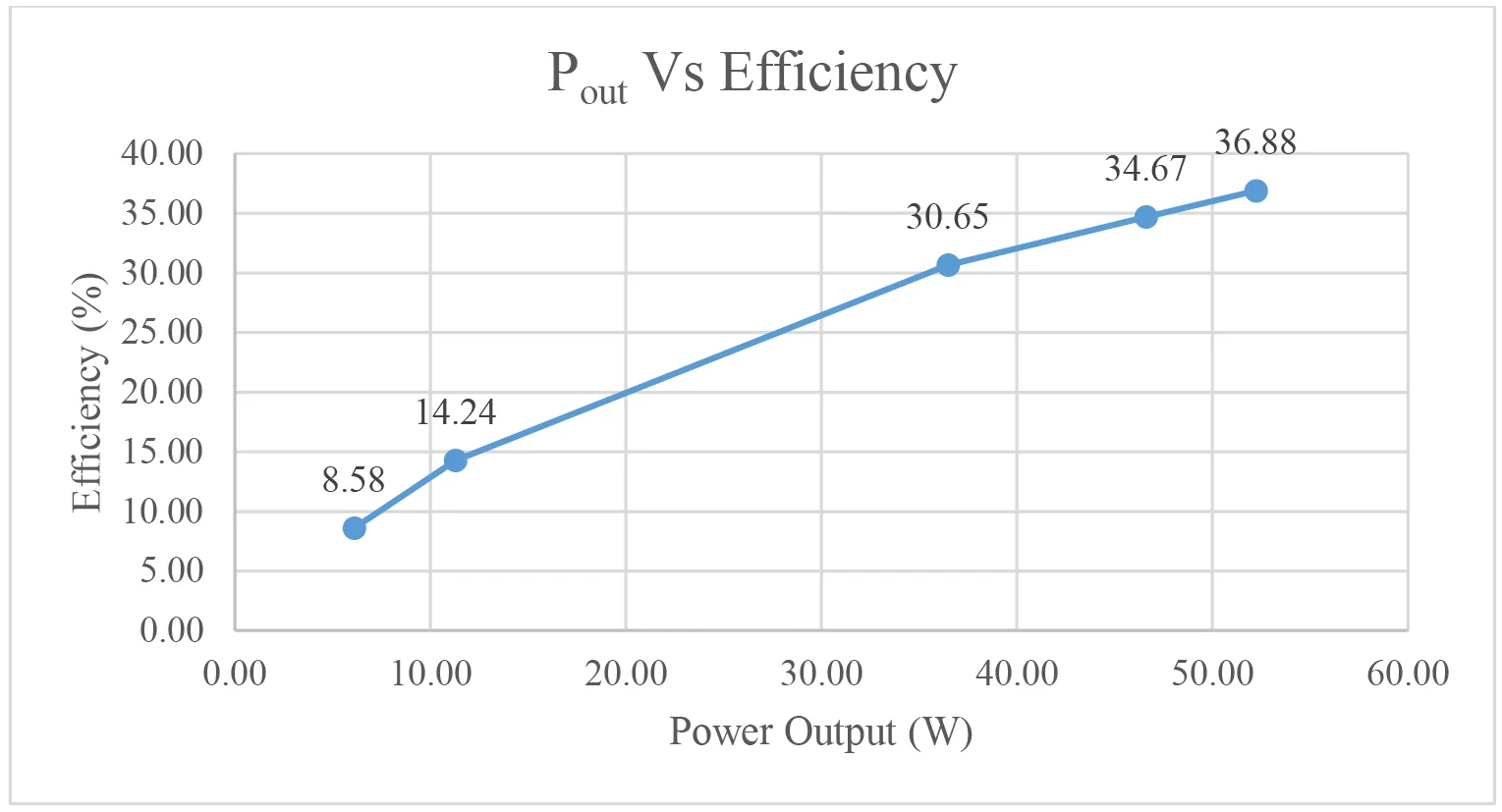
WPT System efficiency at minimum coupling coefficient.

The resulting efficiency is analysed to find the power loss in the system. Power loss analysis is performed to see the source of loss that reduces efficiency. Analysis is performed in the condition of maximum coupling coefficient with 50% load. Loss analysis includes MOSFET conduction loss, conduction loss in the inductor, coil, capacitor, and diode forward voltage loss. Parasitic resistance and current in the compensator inductor, capacitor, and coil, both on the transmitter and receiver side, are measured to calculate the conduction loss. After the data are collected, the calculation for loss analysis is conducted. The input power of the system is 56.64 W, with an output power of 38.51 W, indicating a system loss of 18.33 W. Table 6 shows the calculation results and is visualized in Fig 12. The total power loss is 17.44 W based on the calculation, which is close to the measurement results.

**Table 6:** The loss analysis with the maximum coupling coefficient and 50% load.

	Parasitic Resistance (Ω) of Components									V_F_ (V)
	C_p1_	C_p2_	L_p1_	L_1_	L_2_	C_s2_	Cs_1_	L_s_	Q_1-4_	D_1-4_
	0.022	0.1	0.28	1.21	1.12	0.1	0.022	0.127	0.008	0.6
I_rms_ (A)	0.14	2.66	2.8	2.66	1.8	1.8	0.14	1.8	2.36	1.59
P (W)	0.000	0.324	2.195	8.561	3.629	0.324	0.000	0.411	0.045	1.903
P_total_ (W)	17.488									
P_in_ (W)	56,64									
P_out_ (W)	38,51									
P_loss_ (W)	18,13									

**Fig 12:**
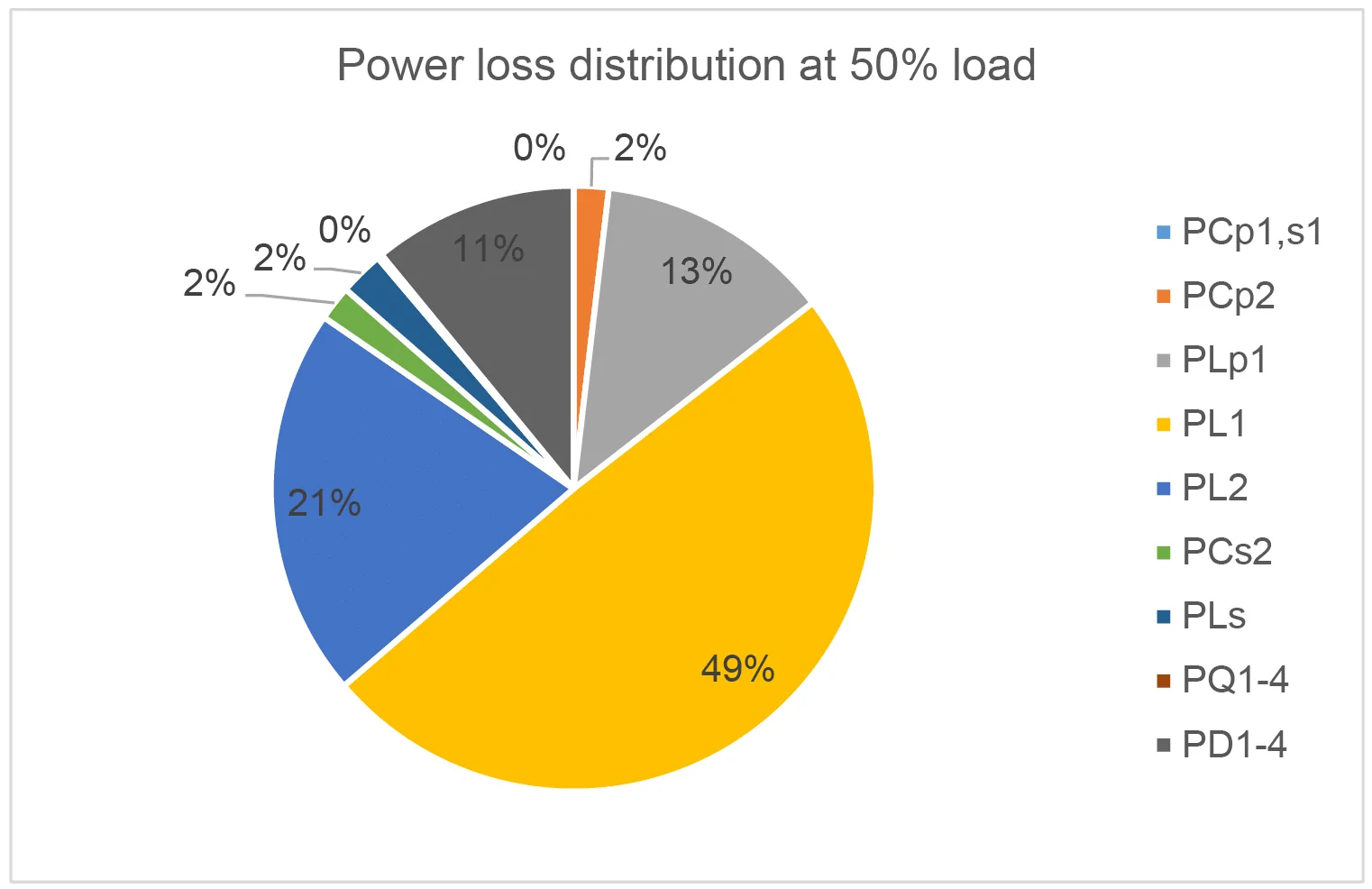
Power loss distribution at the key components.

The largest loss occurs at the transmitter coil, with 49% of the contribution, followed by the receiver-side coil, with 21% of the contribution. The total contribution of coils is 70% of the total loss, while the combined contribution of compensation capacitors is only 3.7%. These values should be a concern when a higher power application is required. It is very important to keep the coil’s total resistance low and the effective current at its optimal value to achieve a more efficient system. As a mitigation, the selection of the coil wire diameter and the reduction of the circulating current in the coils should be considered in the design stage. The remaining power loss can be caused by the IC power consumption, resistance of the PCB tracks, the losses due to the parasitic capacitances of the MOSFET, and the reverse recovery loss of the diode. The analysis shows the biggest loss happens in the transmitter coil with the value of 8.56W. The energy produced due to the losses causes the temperature rise. However, due to the size and the construction of the coil that is placed in the open air, the heat can be transferred directly to the open air. Therefore, no excessive thermal rise was observed during the experiment, which shows the thermal stability of the coils.

The results presented here are limited to the testing scenarios that were explained previously. In the practical operation, the battery charging should have other features such as closed loop control, so that the charger is capable of operating with Constant Voltage (CV), or Constant Current (CC) mode to charge the battery. This can also be done by using a post-regulator circuit and treating the presented WPT system here as the open-loop stage.

## Conclusions

The data acquired shows that in 50–100% load conditions, until a certain limit, the designed WPT system can maintain its efficiency under coil gap and misalignment. Maximum efficiency of the system has reached 69,19% in the lowest coil gap and 2 cm misalignment, conversely lowest efficiency 8,58% in the furthest coil gap and misalignment (8 and 6 cm) with coil slope 30˚.

The WPT system using double-sided LCC has proven successful in transferring power using a wireless method to the receiver side with regulated voltage output by changing the switching frequency. The experiment shows a designed system capable of maintaining a 24 V voltage output under variation of coil gap, misalignment, and slope by changing switching frequency in the range of 63–76 kHz. In conclusion, the WPT system with double-sided LCC compensation is suitable for a farming robot.

The results we obtained are limited to the implemented case. The improvement in the future can be done by implementing the closed loop control system that capable to charge the battery at the required voltage and current level. This could be by adding a post-regulator as the backend of the system.

## Supporting information

S1 Data Double side LCC WPT experimental data. (XLSX)
